# Whole-Genome Sequencing-Based Population Genetic Analysis of Wild and Domestic Rabbit Breeds

**DOI:** 10.3390/ani15060775

**Published:** 2025-03-09

**Authors:** Zsófia Fekete, Zoltán Német, Nóra Ninausz, Péter Fehér, Mátyás Schiller, Maher Alnajjar, Áron Szenes, Tibor Nagy, Viktor Stéger, Levente Kontra, Endre Barta

**Affiliations:** 1 Department of Genetics and Genomics, Institute of Genetics and Biotechnology, Hungarian University of Agriculture and Life Sciences, Szent-Györgyi A. u. 4, H-2100 Gödöllő, Hungary; zsofia.fekete@uef.fi (Z.F.); nora.ninausz@gmail.com (N.N.); feher.peter.arpad@uni-mate.hu (P.F.); matyasschiller@gmail.com (M.S.); alnajjar.maher@uni-mate.hu (M.A.); nagy.tibor2@uni-mate.hu (T.N.); steger.viktor@uni-mate.hu (V.S.); 2 Department of Environmental and Biological Sciences, University of Eastern Finland, Yliopistokatu 2, 80100 Joensuu, Finland; 3 Department of Pathology, University of Veterinary Medicine Budapest, Dóra major, H-2225 Üllő, Hungary; nemet.zoltan@univet.hu (Z.N.); szenes.aron@univet.hu (Á.S.); 4 Department of Biochemistry and Molecular Biology, Faculty of Medicine, University of Debrecen, Egyetem tér 1, H-4032 Debrecen, Hungary; 5 Bioinformatics Core Facility, Institute of Experimental Medicine, Hungarian Research NetworkSzigony utca 43, H-1083 Budapest, Hungary

**Keywords:** rabbit, population genetics, population genomics, breeding, domestication

## Abstract

The European rabbit exists in the wild and has several highly bred domesticated forms. There are well-separated wild European rabbit populations, and intensive breeding has resulted in various forms and utilizations. In this work, we aimed to carry out an extended WGS-based population genomics study on several wild European rabbit populations and selected breeds. Utilizing multiple methods, we showed that although domestic and wild populations were clearly separated, there was evidence of admixture between them in France and Hungary. The populations showed various levels of inbreeding, with one of the Hungarian subpopulations having excess runs of homozygosity. We identified numerous variants fixed in either domestic or wild animals, two of which were found to be fixed at different alleles in the two populations. Some putatively selected regions did not overlap with any known genes in the rabbit genome, suggesting some importance to these intergenic sites. The enrichment of selected regions in certain types of transcription factor binding sites suggests a possible role for these regulatory elements during domestication. In addition, the new high-coverage rabbit whole-genome sequences may provide helpful material for further population genetics analyses.

## Simple Summary

The European rabbit today exists in both its wild form and in several domesticated forms, which makes it an excellent candidate for studies of domestication. However, these wild and domestic populations may interbreed as well. In our study, we investigated this possible interbreeding and relationships between various breeds and populations of wild animals. We also scanned for the presence of genomic signatures of selection during domestication. Our findings show that there is indeed an admixture between wild and domesticated rabbits. We have also found not only genes but regulatory regions that could have played a role in the changes caused by the domestication process.

## 1. Introduction

Rabbits (*Oryctolagus cuniculus*) are the only members of the Oryctolagus genus and are widely known not only as game animals, but have also gone through extensive domestication, resulting in many agricultural and pet breeds. The spread of rabbits from their native range in the Iberian Peninsula has been greatly aided by humans. The species is currently endangered in its original habitat on the Iberian Peninsula (IUCN Red List https://www.iucnredlist.org/species/41291/170619657, accessed on 3 May 2024), where its population has been declining since the first half of the 20th century [1]. Delibes-Mateos et al. (2008) [2] reviewed the role and status of rabbits as keystone species in Mediterranean ecosystems in detail. In numerous areas of the world where rabbits were introduced by humans, they are considered an invasive species [3,4,5].

The existence of rabbits in wild, feral, and domestic forms provides an ideal system not only to study the process of domestication, but also to consider impacts of their contact in the wild resulting in anthropogenic hybridization. Studies in other species highlight two major concerns: hybrids outcompeting wild populations [6] and the sheer amount of crossbreeding irreversibly changing the wild population [7,8]. There are also examples where traits of domestic animals are positively selected in wild populations, thus contributing to their survival [9,10]. Rabbits are primarily bred for their meat, fur, and wool, but have also become an important model system to study disease and in therapeutics. Rabbit domestication is relatively recent, regardless of whether we consider the earliest association with humans or the start of deliberate breeding and selection (1.5–2 thousand years ago [11,12]). Domestic rabbit populations experienced major bottlenecks multiple times. One of these events occurred during the domestication process itself, whereas the other occurred at the breed formation stage, greatly reducing genetic diversity compared with that of wild populations [11]. 

Regarding the nature of rabbit domestication, Carneiro et al. [13] noted the polygenic nature of it, which is in agreement with findings in several other domesticated species [14,15]. Changes during rabbit domestication were likely caused more by shifts in allele frequency than by the emergence of new variants [13]. Many genes and related pathways identified in rabbits include genes related to coat color and structure [16,17] and body size [18,19]. Many changes affected the nervous system and were sensory- and behavior-related [13]. However, some of these changes were related to altered levels of gene expression without the genes themselves containing either new or alternate alleles with significantly changed frequency [20]. Physiological changes in the brain architecture of domestic rabbits have also been noted [21]. We also have to note that most of these studies were designed to detect selection within or between domestic breeds without investigating their relationship with wild populations [22]. In this study, we aimed to investigate at the genomic level both the state of wild populations and the potential admixture of these with domestic animals. After establishing the relationships between populations and evaluating genetic admixture, we analyze the differentiation and selection between domestic and wild rabbits. Within the putatively selected regions, we also investigate the presence of transcription factor binding sites (TFBS) and the severity of mutations in coding sequences.

## 2. Materials and Methods

### 2.1. Sample Collection and DNA Isolation

The samples we collected and sequenced included 14 individuals from 2 wild rabbit populations from Hungary, which were sampled from two locations (N = 6 and N = 8 individuals), and rabbits of domestic breeds, including Rex (N = 5), Thuringian (N = 1), Californian White (N = 3), Hycole (N = 10), Zika (N = 6), and New Zealand White (N = 3).

Blood and ear tissue samples of 28 domestic rabbits were collected from commercial rabbit farms. Blood samples were taken from the ear veins of live animals and collected in EDTA-containing tubes. Ear tissue samples were collected from animals slaughtered for meat production. The samples were stored at −20 °C until the start of DNA isolation.

Approximately 20 mg of ear tissue per sample was used for DNA extraction with the MagCore Genomic DNA Tissu Kit (RBC Bioscience Corp., New Taipei City, Taiwan) according to the manufacturer’s recommendation, using a MagCore HF16Plus Automated Nucleic Acid Extractor robot (RBC Bioscience Corp., Taiwan) instrument. The elution volume was 200 μL. Genomic DNA from blood samples was isolated using the MagCore Genomic DNA Whole Blood Kit (RBC Bioscience Corp., Taiwan) according to the manufacturer’s instructions. A blood sample volume of 400 μL and elution volume of 200 μL were used with the same instrument.

The concentration of the DNA was measured with a NanoDrop ND-1000 (Thermo Fisher Scientific, Waltham, MA, USA).

### 2.2. Sequencing and Public Data Collection

We analyzed whole-genome sequencing (WGS) data from 98 samples in total, of which 56 were public data downloaded from the NCBI SRA, and 42 samples (14 wild, 28 domestic) were sequenced for this study.

Library preparation and sequencing of the latter samples were performed by Novogene (Singapore), following a standard WGS protocol (paired-end, 150 bp read length), on an Illumina NovaSeq 6000 system (Illumina, Inc., San Diego, CA, USA) to an average depth of 35.4 (± 6.37).

All public rabbit WGS data that were available on 2022.05.30 on the NCBI SRA were checked for suitability. The samples obtained from the NCBI SRA contained WGS data of individual (New Zealand White, Japanese White, Watanabe [23], Netherlands Dwarf [24], Angora) and pooled (wild French and Iberian populations, Belgian Hare, Dutch, Flemish Giant, French lop, Champagne d’Argent [13]) samples. The average coverage was 9.5, with a minimum coverage of 3.9 and a maximum coverage of 14.3. Any WGS data of rabbits of unknown or otherwise unidentifiable breed or origin due to missing metadata were excluded. A list of the selected SRA accessions and samples collected and sequenced for this study is also provided in Table S1.

### 2.3. Quality Control and Variant Calling

Following initial quality control with FastQC v0.11.7, Illumina adapter sequences and low-quality reads were removed using Trimmomatic v0.36 [25] (sliding window size 30, step size 4, minimum read length 50, leading and trailing cutoff under a quality score of 28). The trimmed data were aligned to the OryCun2 reference genome [13] using the Burrows–Wheeler Alignment Tool (bwa mem v0.7.17 [26]) with default settings. After alignment, we followed the best practices protocol recommended by the Genome Analysis Toolkit (GATK v4.1.8) [27,28]. We used the hard filtering method of GATK’s VariantFiltration due to the lack of a high-quality known rabbit SNP (Single nucleotide polymorphisms) database. Variants were filtered for minimum allele frequency (0.001) and allele count (biallelic: min. 2, max. 2) in PLINK 1.9 ([29] www.cog-genomics.org/plink/1.9/). PLINK 1.9 was also used for LD pruning (option --indep-pairwise 50 10 0.1).

We only used biallelic SNP and indel variants. Missing calls were not allowed, and noninformative sites (all homozygous reference calls) were excluded. Only sequences of primary chromosome assembly were included, as unplaced scaffolds are often too short for meaningful analysis.

Called variants were annotated for effect and impact severity using SNPEff [30], based on the Ensembl (release 104) [31] gene annotation.

### 2.4. Detecting Population Structure and Inbreeding 

Eigenvector values for the principal component analysis (PCA) were calculated in PLINK 1.9 [27]. We constructed a maximum likelihood tree from the SNP data using the SNPhylo (v. “20180901”) [32] pipeline.

We used ADMIXTURE v1.3 [33] to check for possible interbreeding between wild and domestic populations. The cross-validation error was calculated for 1 to 12 populations. Admixture was calculated for K values with the two lowest cross-validation errors, K = 5 and K = 7, as well as for K = 6. We used Treemix v.1.13 [33] software to confirm the admixture of Hungarian samples using samples from geographically close areas that were sequenced to a high coverage, with the exception of the outgroup sample of *Oryctolagus cuniculus algirus*. The two batches of wild Hungarian samples were considered two separate populations because of the different sampling locations. Five hundred bootstrap replicates were used. We ran D (ABBA-BABA) and f4 tests as implemented in the ADMIXTOOLS suite and the admixr R package [34,35].

The level of inbreeding in wild Hungarian rabbit populations was assessed by looking for runs of homozygosity (ROH). Pooled WGS samples were excluded from ROH counts. The ROH were detected with PLINK 1.9 and then further categorized, quantified, and visualized with custom-made bash and R scripts. The minimum length of homozygous sequences considered an ROH was set at 100 kbp. Above this, we divided ROHs into further length categories, with the highest category containing any ROH over 1 Mbp.

### 2.5. Selected Genes in Domestic Breeds Based on Wgs Data of Unadmixed Individuals

Genomic regions under selection were located using a combination of composite likelihood ratio (CLR), nucleotide diversity (π), and fixation index (Fst) analyses. The CLR test was performed with the help of SweepFinder2 [36]. SweepFinder2 calculates an empirical background frequency spectrum prior to testing, from which the likelihood of the null hypothesis is then calculated. An approximate 10 kb spacing between test sites was set.

For the calculation of Fst values, we used the method of Weir and Cockerham [37], as implemented in VCFtools 0.1.17 [38]. VCFtools was also used to calculate nucleotide diversity. In both cases, a sliding window approach was used with a window size of 10 kb and a step size of 5 kb to maintain a scaling similar to that of the CLR test.

The extreme ends (99th percentile) of all test results, i.e., the highest values for Fst and CLR and the lowest for nucleotide diversity, were considered potential candidate regions under selection. The overlap of the candidate regions from each test was determined using Bedtools intersect [39]. Neighboring windows that were candidates for selection were then merged, with a maximum distance of 10 kb between windows passing the threshold.

The resulting regions were intersected with Ensembl annotation (release 104) [31] to identify overlapping genes. Liftover [40] was used to map transcription factor binding sites (TFBSs) from the human ChipSummitDB database [41] to the rabbit genome. The potential TFBSs were then intersected with the candidate regions.

We conducted gene ontology tests on genes identified with at least two methods to test for statistical overrepresentation using Fisher’s exact test. The Panther GO-Slim biological process, molecular function, and pathways [42,43] categories were checked. Mouse and human orthologs for rabbit genes were obtained from Ensembl BioMart (release 109) [44].

## 3. Results

### 3.1. Sequencing and Data Collection

We gathered whole-genome sequencing data from 98 samples of *Oryctolagus cuniculus*, of which 56 were publicly available from the NCBI SRA, and 42 samples were collected and sequenced for this study.

After quality control, alignment, variant calling, and filtering of the calls, 27,524,641 variant sites were retained, as they satisfied all our criteria. These sites included 21,863,765 SNPs and 5,660,876 indels. We further removed variants with any missingness (only sites genotyped in all samples were allowed) or high LD. After all the filters were applied, we retained 2,780,336 variants (Table 1).

### 3.2. Population Structure and Phylogenetic Tree

**Figure 1:**
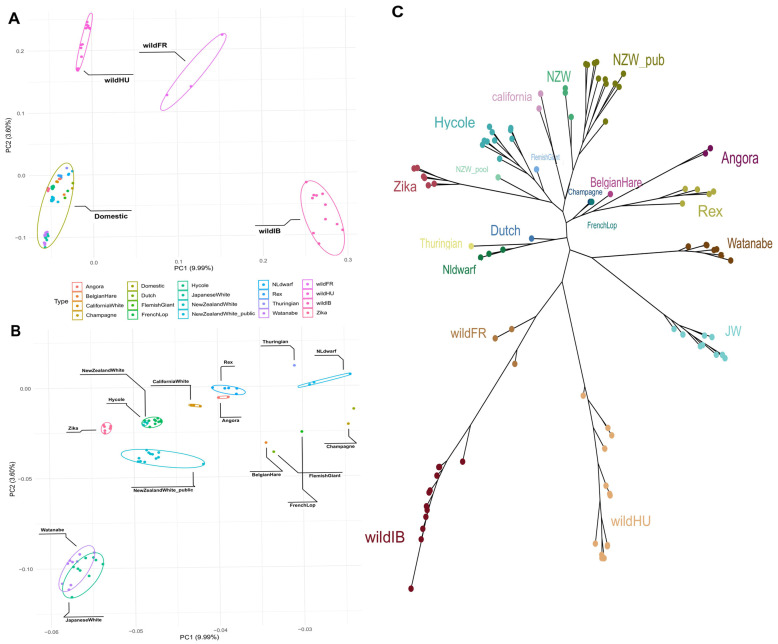
Principal component analysis and phylogenetic tree. (**A**) Principal component analysis plot of all samples used. A clear separation between the domestic breeds and the different wild populations can be seen. (**B**) Subgroups within the domestic rabbits also show separation. (**C**) Maximum likelihood tree constructed based on the called variants. Most of the tree aligns well with what is seen in the PCA. The main difference is that the wild Iberian and French samples are on the same branch and are much closer to each other.

The PCA results clearly revealed separate groups of wild and domestic populations (Figure 1A). The French (wildFR), Hungarian (wildHU), and Iberian (wildIB) populations clearly differed in terms of their separation from domestic breeds. The difference between domestic breeds was also noticeable but far less pronounced than that between each of the wild populations and the wild and domestic populations (Figure 1B). Among the domestic breeds, Japanese White and Watanabe rabbits seemed somewhat further apart from the other domestic breeds, which were generally European and North American in origin (EU/NA).

The maximum likelihood tree constructed from the filtered SNP data revealed branches separated by breed (Figure 1C). While the wildFR and wildIB populations were placed on the same branch, the third wild population, wHU, was separated similarly to the PCA results, while keeping the same root as the other wild populations. Once again, the two Japanese breeds grouped closer to each other and were more separated from the other domestic breeds. The remaining domestic breeds clustered into several smaller groups. Notably, these groups can be separated by breed purpose: New Zealand White, Californian, Hycole, and Zika rabbits are bred mainly for their meat, whereas Rex and Angora rabbits, even though they are both favored as pets as well, both have unique qualities to their fur. The Thuringian, Netherlands Dwarf, and Dutch rabbits are small-bodied breeds kept as pets. One New Zealand White WGS sample, a pooled sample acquired from the SRA, was grouped with the Zika rabbits from our samples.

### 3.3. Admixture of Rabbit Breeds and Populations

The results of the cross-validation (CV) test of ADMIXTURE [33] suggest the best value at K = 5 (0.19826) as the most likely number of ancestral populations (Figure S1). Interestingly, while the CV error was greater at K = 6 (0.21476), in the case of K = 7 (0.2009), it was lower again, closer to the level of K = 5. At K = 6, ADMIXTURE grouped the individuals fairly differently, separating one more wild ancestral population while the other two separated more domestic ones. However, with the higher cross-validation error at K = 6, it was less likely to represent the actual population structure than either K = 5 or K = 7.

**Figure 2:**
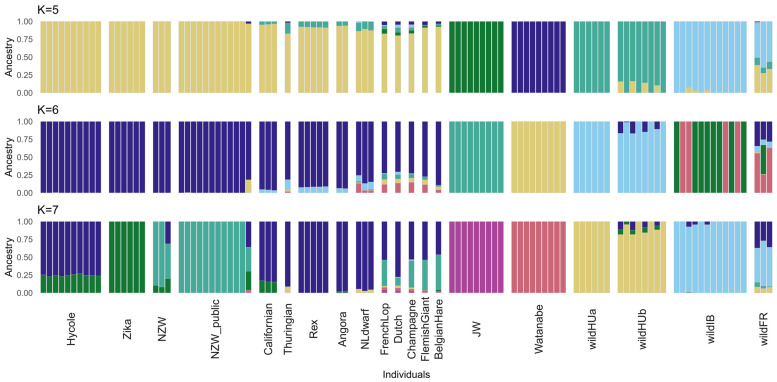
Results of the ADMIXTURE test. The ADMIXTURE results are shown for the number of populations with the lowest cross-validation (CV) error and the intermediate K value of 6. K = 5 and K = 7 had the lowest CV error values (0.19826 and 0.20090, respectively), indicating the most accurate number of populations. The analysis revealed that the wild French and some wild Hungarian samples exhibited admixture with at least one type of domestic breed, suggesting a history of genetic exchange. With K = 5 populations assumed, most domestic rabbits are grouped together, which then becomes more split with K = 7, indicating further genetic diversity within the domestic breeds. The Japanese White and Watanabe breeds are consistently separate from the other domestic samples, suggesting their unique genetic profiles.

The level of admixture was calculated using the K values of 5, 6, and 7. The groups separated at K = 5 were wild Hungarian (wildHU), wild French and Iberian (wildFR/IB), the two Japanese breeds, and the remaining domestic breeds. Interestingly, the Japanese White and Watanabe breeds were also separated by ADMIXTURE, whereas the remaining domestic breeds were grouped together until the calculation with K = 7. In either case, the results revealed the possible interbreeding of wild and domestic rabbit populations in both Hungary and France. Separating the wildHU samples into two subpopulations according to sampling location revealed admixture within only one of these groups (Figure 2).

**Figure 3:**
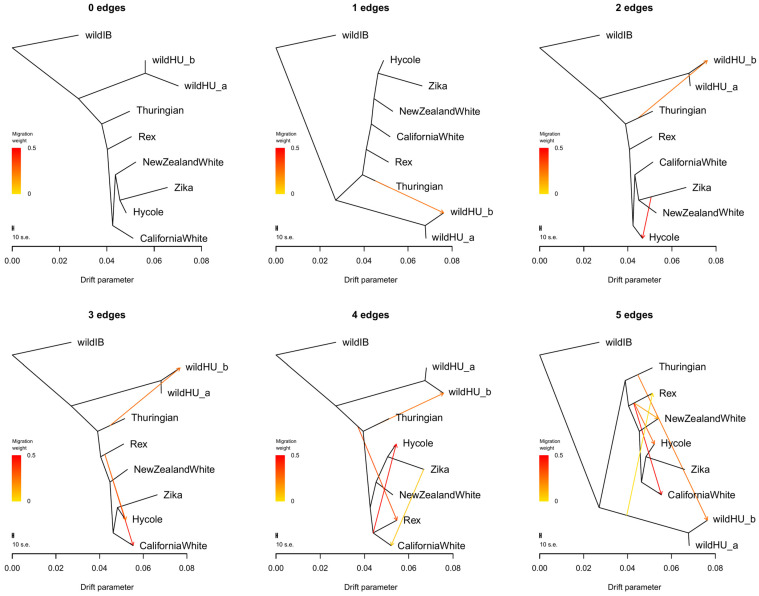
Results of Treemix runs. Treemix plots for different numbers of assumed migration events between populations, without sample size correction. These plots provide valuable insights into the historical gene flow between populations, shedding light on the complex patterns of genetic exchange. Notably, there is a consistent migration edge from the Thuringian breed to the wild Hungarian samples, confirming the admixture of the wildHUb subpopulation with domestic animals. All other events show gene flow between domestic populations, indicating a complex pattern of genetic exchange within the domestic rabbit population. The residual error values, shown in Supplementary File, Figure S2, further validate the accuracy of our findings, enhancing the reliability of our research.

Further examination of possible admixture events between the wildHU and domestic breeds in Treemix software (Figure 3) confirmed these results. Once again, wildHU was separated into wildHUa and wildHUb subpopulations. The results revealed admixture between Thuringian rabbits and the wildHUb population, with the direction of migration pointing domestic to the wild at any number of assumed migration edges tested. However, despite the good fit, we must consider that we had only one Thuringian sample. The Thuringian sample shares its main ancestry with the other domestic breeds, making it possible that the introgression is from other breeds, despite the results inferred by Treemix. The residual fit from each tree is shown in Figure S2. We further controlled these results by calculating the D and f4 statistics, which also supported the wildHUb/domestic admixture (Figure S3).

### 3.4. Runs of Homozygosity and Inbreeding

**Figure 4:**
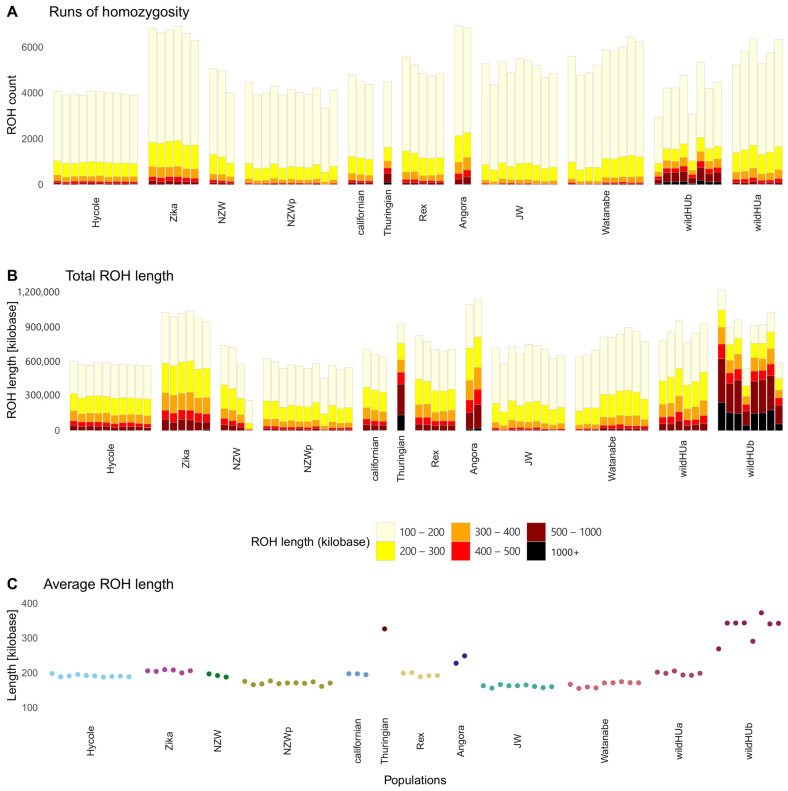
Runs of homozygosity in the surveyed populations. Results of runs of homozygosity (ROH) tests, which were run on individual samples. (**A**) Total ROH count: columns colored by the length of runs make up the total amount. (**B**) Total ROH length in kilobases, colored by the length of each run, shows the total ROH length composition. The total length of ROH sequences in the Thuringian breed and wildHUb individuals are similar to those in the other populations, but more ROH sequences are found in longer runs. (**C**) Average ROH lengths across populations, with the Thuringian and WildHUb animals showing the highest averages.

The extent of inbreeding was analyzed using the run of homozygosity (ROH) test. Pooled samples were excluded from this analysis. The results (Figure 4) revealed that samples of the two wildHU subpopulations presented different levels of inbreeding: the wildHUb population presented a lower number of ROH regions, which were longer, which usually indicates more recent inbreeding. The ROH regions with the greatest total length were found in the wildHUb population and in the Zika and Angora rabbits (Figure 4).

### 3.5. Selection in Domestic Rabbits

To avoid interference, we excluded admixed wild rabbit samples from the domestication tests (i.e., four of the wild Hungarian and all of the wild French rabbit samples).

The overlaps of regions with the extreme-most values (99th percentile) of each test provided 46 potentially genetically selected regions, in which some long genes, namely, *SCN9A, POLR1B, LCORL, SIPA1L2, MAP3K21, PCNX2*, and *SCN7A*, spanned more than one region (Supplementary Table S2). Some of these genomic regions also overlapped with multiple genes (max. 3), whereas others contained none. A total of 27 genes, including 23 protein-coding genes and 4 long noncoding RNA genes, were found to overlap with 32 candidate regions. Fourteen of the candidate regions did not overlap with any known genes. For a summary of the overlapping regions and their gene content, see Supplementary File Table S2.

**Figure 5:**
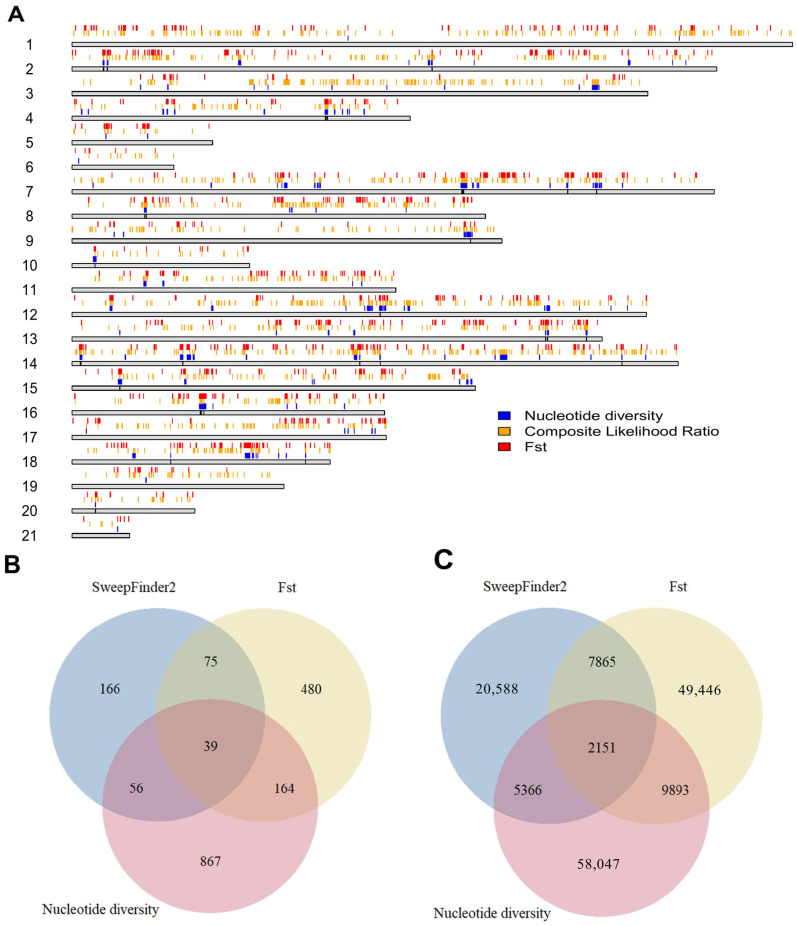
Regions that tested positive for selection and their features. (**A**) Top candidate regions with each method used for tests of selection. The gray bars represent the length of each chromosome, with the colored regions above them showing the regions in the 99th percentile of each test. (**B**) Gene contents of genetically selected regions by test and their overlaps. (**C**) Putative transcription factor binding sites in each selected region and their overlaps.

We also identified a total of 1,985,753 putative transcription factor binding sites on primary chromosomes based on the ChipSummitDB database, 1170 of which were found in candidate regions. Among these, 93 were found in regions that did not overlap with genes. The positions of the selected regions in the genome, as well as the numbers of genes and TFBSs, are shown in Figure 5.

Gene ontology tests did not reveal any significant enrichment in any of the categories evaluated. However, based on Fisher’s exact tests, seven types of transcription binding sites were enriched in the selected candidate regions (Table 2).

Further analysis of the candidate regions revealed several highly differentiated variants. Within these regions, there were 253 variants with Fst values greater than 0.9, and 983 of them had Fst values greater than 0.75 between the domestic and wild populations. Among these highly differentiated variants, many (683 of 983, 69.48%) were found to be completely fixed in domestic animals, but only eight appeared to be completely fixed in the wild populations, despite having a large number of samples from various breeds of domestic animals and a limited pool of wild samples. Only two of the variants were completely fixed at different alleles in both the wild and domestic populations. These two variants were found in gene ENSOCUG00000008635, an ortholog of the human and mouse *MAN1C1* genes (Ensembl).

Approximately half of these highly differentiated variants were found to overlap with genes (473 of the 983 Fst values over 0.75 were in genes, 142 (92% of 253) of which were over 0.9), and only a few of them overlapped with exons (9) or predicted TFBSs (15 in total, 8 over 0.9). A summary of these highly differentiated variants and affected genes can be found in Table 3.

The gene affected by the greatest number of highly differentiated variants was *CFLAR*, with a total of 61 variants with Fst values over 0.9 and 132 over 0.7. The second was a novel gene, ENSOCUG00000038656, with 131 variants, of which 40 had Fst values over 0.9. According to the Ensembl database, this gene has no known orthologs. The genes with highly differentiated exonic variants included the protein-coding genes *PPP2R3A, BCL6, CFLAR*, and *SCN7A* and a long noncoding RNA (lncRNA) gene (ENSOCUG00000034993).

## 4. Discussion

While there are various studies available on domestic rabbit breed formation [18,19,45,46], few have delved into the domestication process itself, especially those utilizing high-coverage whole-genome sequencing of various breeds and populations. Other approaches, such as reduced-representation sequencing and other genotyping methods, may be more affordable than high-coverage whole genome sequencing, but they limit genetic information to certain parts of the genome. This may exclude potentially important regions, as sequence variations responsible for different genetic traits can be scattered throughout the whole genome. A hint about the importance of these regions may provide pointers for further studies.

### 4.1. Population Structure and Diversity

Our PCA and phylogenetic (Figure 1) analysis showed consistent results in the grouping of populations. Interestingly, the Rex and Angora rabbit breeds grouped on the same branch in the phylogenetic tree despite their coats having been selected for different traits. Nonetheless, they are both primarily bred for the quality of their fur, even though they have different qualities and the regulation of hair traits is highly complex [16,44]. The rest of our samples were from either meat-producing or laboratory-use breeds, apart from the dwarf breeds and the Dutch rabbit, the latter two being mostly kept as pets.

Another notable exception to the consistency was the pooled New Zealand White (NWZ_pooled) sample, which was distinct from the other NZW samples, considering the public and our samples alike, which could be explained by potential crossbreeding with other breeds or strong population structure within the breed. Owing to the nature of sample pooling and the lack of detailed information on the samples included, it is not possible to determine the exact cause of this difference.

The Japanese White and Watanabe breeds were separated by ADMIXTURE at the lowest cross-validation errors, whereas the remaining domestic breeds were mostly grouped together (Figure 2). Although the Watanabe breed was fairly recently derived [45] from the Japanese White breed, there was a severe bottleneck between the two, as Watanabe rabbits were specifically selected to be susceptible to hyperlipidemia. Our results are in line with the work of Xie et al. [45].

While the maximum likelihood tree and the principal component analysis plot were also not completely aligned in the placement of the wild populations, we must consider that our first two principal components explained only a limited percentage of all variance. This, coupled with the ADMIXTURE results showing the extent of similarity the French samples shared with the domestic breeds, could explain the different placements. Arguably, the placement on the maximum likelihood tree is closer to reality, as French wild rabbits are most likely descendants of Iberian wild rabbits, which connection is confirmed by the ADMIXTURE results, and an ancestral population of most, if not all, domestic breeds [11].

Conversely, the wild Hungarian population seemed distinct from every other wild or domestic group, suggesting a unique history of the population. While one of the subpopulations of wild Hungarian rabbits was clearly admixed with domestic rabbits (Figure 3), this population is kept at the Budapest Zoo with no intention of rerelease and therefore does not interfere further with the genetic pool of free-living wild rabbits. We have no information on where these individuals or their ancestors were originally captured, but there may be wild populations that are currently mixed with domestic rabbits. On the other hand, the sampling location and the living conditions of the zoo population explain the greater degree of inbreeding.

The overall diversity followed a similar pattern to that reported by Magalhaes et al. [47] based on their study of the DQA gene of the major histocompatibility complex in wild Iberian, French, and domestic rabbits. In general, wild rabbits present relatively high levels of genetic diversity compared to domestic rabbits due to the latter being affected by strong bottlenecks during domestication. Even though it does not seem to be a serious concern for free-living wild populations at present, inbreeding and reduced diversity can easily become concerns because they affect fertility rates [48] (Figure 4).

### 4.2. Divergence and Selection of Wild and Domestic Rabbits

The samples of the free-living wild Hungarian population of rabbits and the nonadmixed individuals of the captive population could be used to study the divergence between the domestic and wild rabbit populations. Wild French rabbit samples were excluded from the tests of selection because of their high genetic similarity and likely admixture with domestic animals.

Our tests (Figure 5) of selection and divergence show that, while there were many highly differentiated variants in both genic and intergenic regions, relatively few of them were found in exons of genes, and many of them had a low impact, according to our annotation with SNPEff [46]. In this context, our results are consistent with other studies of domestication in several species, as well as with those performed in rabbits [12]. While most studies have focused on selected genes, we also tested the putatively selected regions for their transcription factor binding site content. As transcription factor ChIP-seq data are still not available for rabbits, we utilized the consensus binding sites in our human ChIPSummitDB database and searched for conserved sites in rabbits. Interestingly, enrichment of certain transcription sites was noted (Table 2) In the case of regulatory elements, even [47] small genetic divergence can result in high phenotypic differences due to altered levels of gene expression [20]. For the analysis of this type of difference, combining genome and RNA sequencing studies might be best.

Our results (Table 3) support earlier findings that domestication is more likely a result of shifts in allele frequency at many loci rather than the emergence of new variants [13]. This is shown in our study by the presence of many highly differentiated variants, of which only two were completely fixed at different alleles.

Many more fixed alleles were found in domestic animals than in wild animals, despite the inclusion of several breeds in our study. Genetic diversity is often lower in domestic animals, as they are purpose-bred rather than naturally selected for fitness and often experience severe bottlenecks during both domestication and breed formation. This is also the case for rabbits [11,47]. There is also a large geographic distance between the wild populations tested from different countries (wildHU and wildIB), possibly contributing to the greater degree of overall observed diversity. In addition to isolation by geographic distance, environmental differences are also a significant factor, as shown by Ziege et al. [49], even over a much shorter distance.

Some of the genes with the greatest allele frequency shifts (Table 3) include *CFLAR*, a neurodevelopment-related gene, and *LCORL*, which is related to body size and development. Other developmental genes were also found to be affected, highlighting the importance of quick growth, large size, and docile behavior in many domesticated animals. *LCORL* was also found to be under selection in other studies of selection in rabbits [18,43]. However, these previous studies focused on breed formation, whereas our study involved a variety of domestic breeds and compared those breeds to wild populations. The latter also explains the limited overlap between the genes identified in our work and those in the papers mentioned previously.

## 5. Conclusions

We have sequenced 42 new rabbit whole genomes with high coverage, which, in addition to this work, may also provide helpful material for further population genetics studies. By combining these with publicly available data, we successfully applied different population genetics methods to better understand the domestication and population-based differences of this species.

In our tests of selection, our results were in line with other studies. These findings support the view that the process of domestication is a multigenic process involving many loci and shifts in allele frequency patterns rather than the emergence and fixation of new alleles. However, the genes detected as selected differed, revealing differences between the processes of domestication and breed formation. We also pinpointed possible genomic regions that might have been involved in evolutionary changes during the domestication of rabbits. Notably, these regions are not necessarily genic regions, suggesting that intergenic regions and elements with currently unknown functions could play important roles. In addition, we found that certain transcription factor binding sites were enriched in the selected regions.

## Figures and Tables

**Table 1:** Samples and variants before and after filtering steps.

Filtering Steps	Quality Filtering	Missingness and Allele Count	LD Pruning
Samples	100	97	97
Variants	67,084,502	27,524,641	2,780,336
SNPs	50,253,306	21,863,765	1,825,434
Indels	18,882,395	5,660,876	954,902
Others (mixed)	1,352,355	0	0
Multiallelic sites	8,344,576	0	0
Multiallelic SNP sites	2,506,463	0	0

**Table 2:** Transcription factor binding sites found in candidate regions under selection.

Total in Genome	Name	Found in Selected	Fisher_Exact p
9610	Atf3	11	0.029718
1796	Bcl6	4	0.02263
46,581	CEBPB	38	0.030488
5	ESR1	1	0.002941
18,173	NFIC	21	0.003292
9848	Rxra	11	0.034464
2302	ZNF143	7	5.07 × 10^−4^

**Table 3:** Highly differentiated variants in selected genes.

Gene ID	Per-Site Fst		Name
	>0.75	>0.9	
ENSOCUG00000001839	72	22	*PPP2R3A*
ENSOCUG00000001843	72	22	*MSL2*
ENSOCUG00000002636	9	0	*FHIP1A*
ENSOCUG00000005429	9	0	*BCL6*
ENSOCUG00000005623	39	0	*SCN9A*
ENSOCUG00000005958	2	1	*ATRNL1*
ENSOCUG00000006058	1	2	*POLR1B*
ENSOCUG00000006802	11	0	*LCORL*
ENSOCUG00000007266	3	0	*SCN1A*
ENSOCUG00000008219	24	5	*POC1B*
ENSOCUG00000008635	36	19	novel
ENSOCUG00000010297	11	0	*SIPA1L2*
ENSOCUG00000010372	8	0	*MAP3K21*
ENSOCUG00000011072	132	61	*CFLAR*
ENSOCUG00000014363	40	0	*PCNX2*
ENSOCUG00000015875	16	8	*RFTN1*
ENSOCUG00000016507	11	3	*ATP2B1*
ENSOCUG00000016916	58	38	*CEP85*
ENSOCUG00000016927	58	38	*SH3BGRL3*
ENSOCUG00000016938	58	38	*UBXN11*
ENSOCUG00000017729	42	0	*SCN7A*
ENSOCUG00000032144	97	33	novel, lncRNA
ENSOCUG00000034322	11	5	novel, lncRNA
ENSOCUG00000034812	3	0	novel
ENSOCUG00000034993	97	33	novel, lncRNA
ENSOCUG00000038656	131	40	novel, lncRNA
ENSOCUG00000039398	5	1	novel

## Data Availability

Sequencing data are available at the NCBI SRA, BioProject ID: PRJNA1118464.

## Supplementary Materials

The following supporting information can be downloaded at: https://www.mdpi.com/article/10.3390/ani15060775/s1, Table S1: Samples used in our study, Table S2: Selected regions and their gene contents, Figure S1: Plot of cross-validation errors by ADMIXTURE; Figure S2: Residual fit of Treemix plots; Figure S3: Results of D and f4 tests of admixture.
