# Supplementary material for: Whole-Genome Sequencing-Based Population Genetic Analysis of Wild and Domestic Rabbit Breeds
Source: Animals (Basel). 2025 Mar 9;15(6):775. doi: 10.3390/ani15060775 (PMC11939179; doi:10.3390/ani15060775)
Supplement: Supplementary file 1 [file animals-15-00775-s001.zip › Supplementary Materials.pdf]

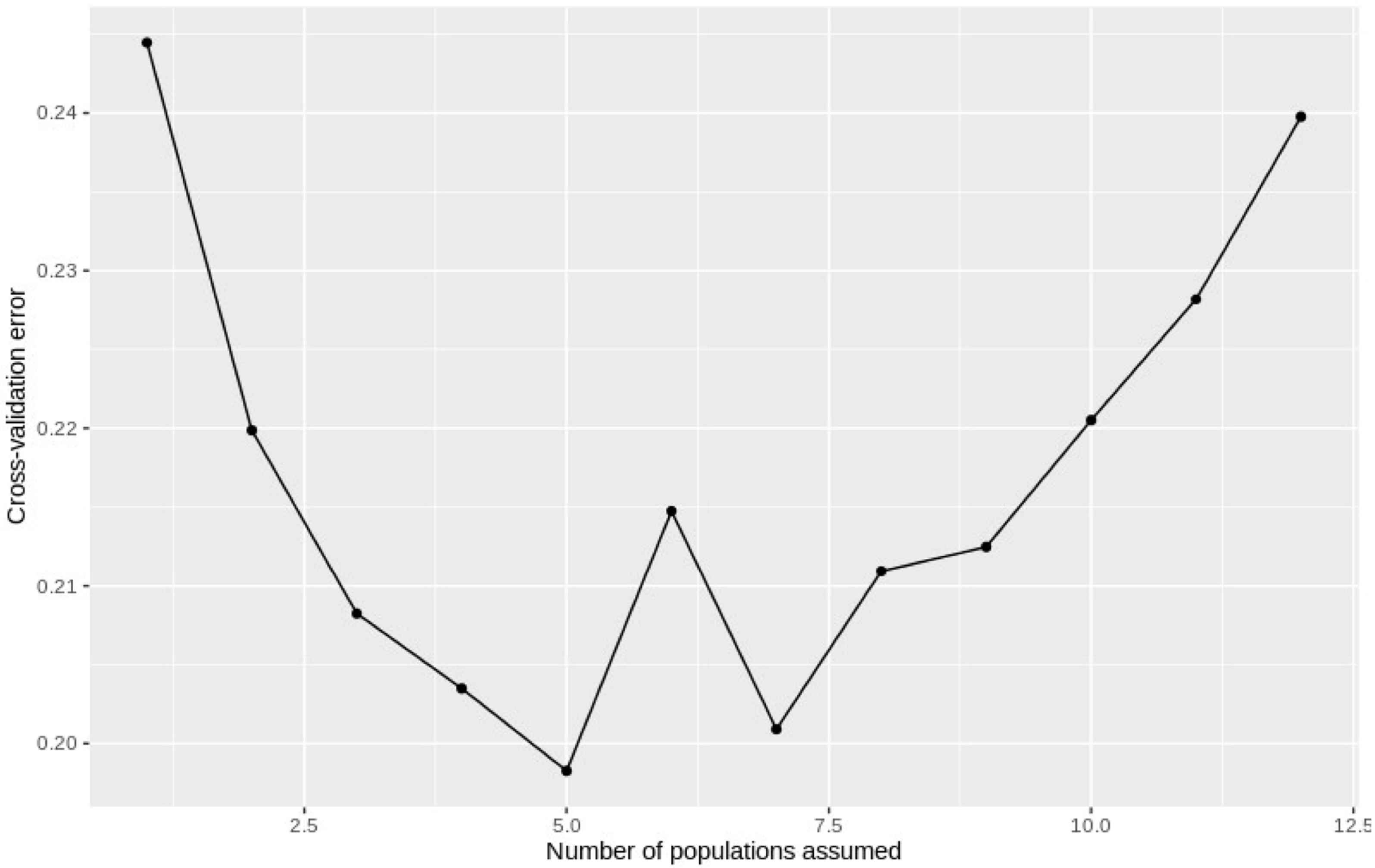

Figure S1. Plot of cross-validation errors by ADMIXTURE

Exact values for each K are as follows:

CV error K=1: 0.24447, K=2: 0.21987, K=3: 0.20824, K=4: 0.20349, K=5: 0.19826, K=6: 0.21476, K=7: 0.20090, K=8: 0.21094, K=9: 0.21249, K=10: 0.22051, K=11: 0.22818, K=12: 0.23977

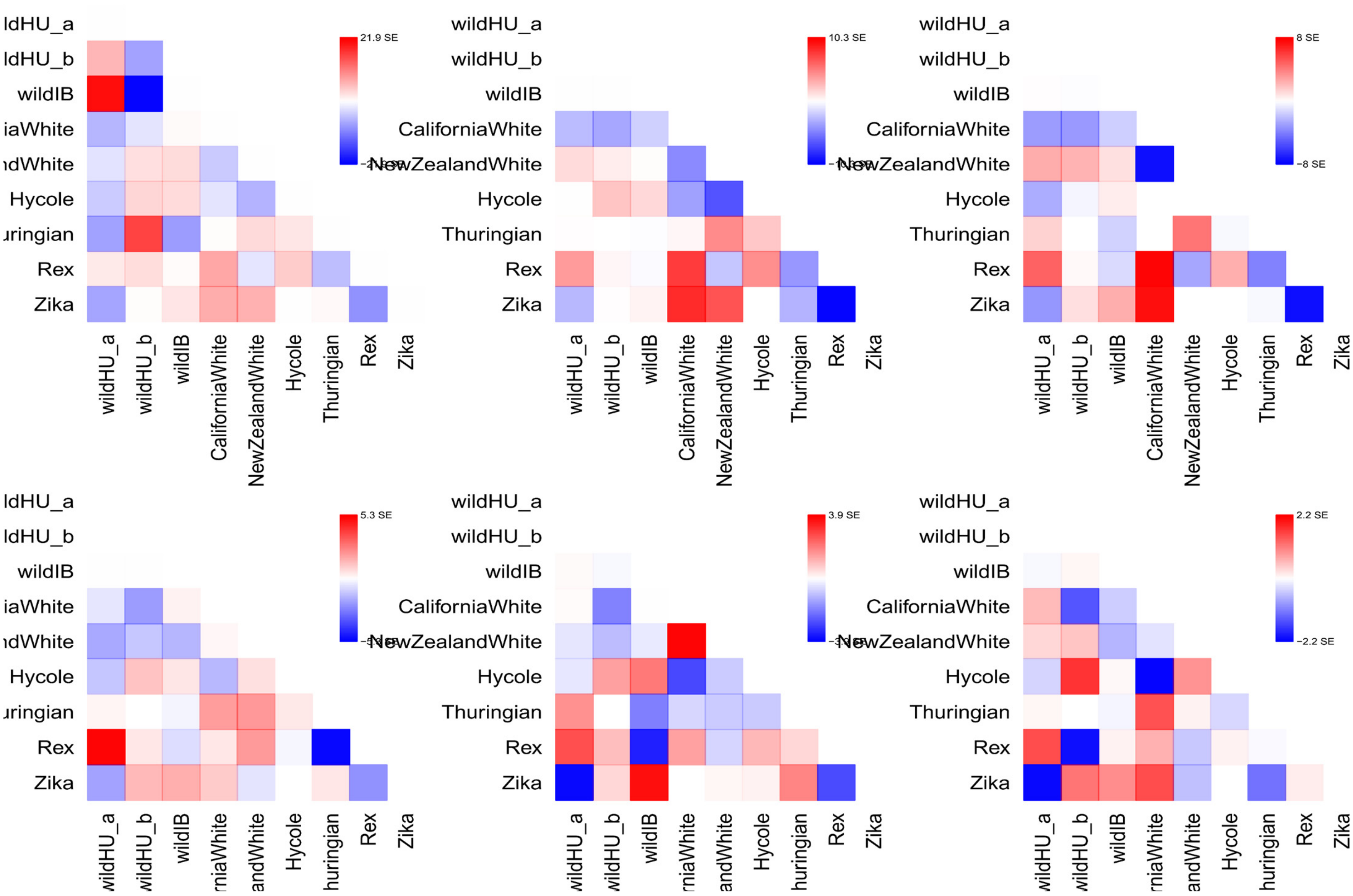

Figure S2. Residual fit of Treemix plots.
Color and color intensity show the direction and relative intensity of the discrepancy between the calculated values and the tree. SE = Average standard error x 10. Positive values mean that the populations are more closely related in the data than in the best fit tree.

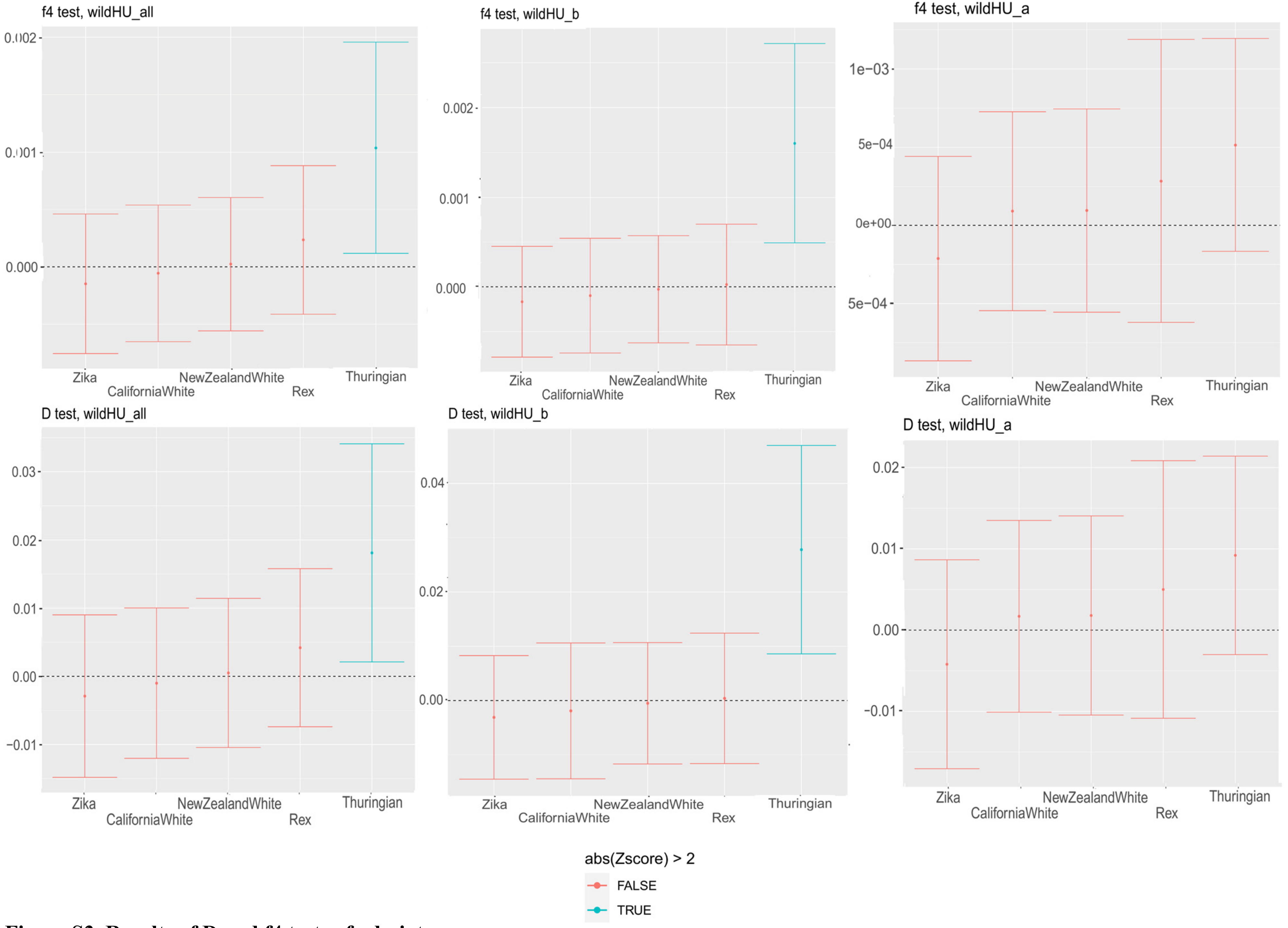

Figure S3. Results of D and f4 tests of admixture.

The test results consistently show admixture with domestic rabbits in one of the wild Hungarian subpopulations. While calculating for all Hungarian samples, the overall results are still positive, however, there is no sign of admixture if we only consider population wildHU_a. The results of these tests are consistent with the results from the ADMIXTURE and Treemix softwares.
